# Complete Genome Sequence of *Mycobacterium* sp. Strain 3519A

**DOI:** 10.1128/genomeA.01565-17

**Published:** 2018-01-25

**Authors:** Amar Bouam, Anthony Levasseur, Maryline Bonnet, Laurence Borand, Charles Van Goethem, Michel Drancourt, Sylvain Godreuil

**Affiliations:** a Unité de Recherche sur les Maladies Infectieuses et Tropicales Emergentes CNRS 6 UMR 7278, IRD198, INSERM U1095, Institut Hospitalier Universitaire Méditerranée-Infection, Aix-Marseille Université, Marseille, France; b UMI233-TransVIHMI/INSERM U1175, Institut de Recherche pour le Développement (IRD) and University of Montpellier, Montpellier, France; c Epidemiology and Public Health, Institut Pasteur du Cambodge, Phnom Penh, Cambodia; d Département Biopathologie Cellulaire et Tissulaire des Tumeurs, Centre Hospitalier Universitaire (CHU) de Montpellier, Montpellier, France; e Laboratoire de Bactériologie, MIVEGEC, Centre Hospitalier Universitaire (CHU) de Montpellier, Université de Montpellier, Montpellier, France

## Abstract

*Mycobacterium* sp. strain 3519A is a nontuberculous mycobacterium isolated from sputum from a Cambodian patient with a pulmonary infection. We report here the first complete 7.3-Mbp-long genome sequence of *Mycobacterium* sp. 3519A with 66.35% GC content, encoding 7,029 protein-coding genes, 50 tRNAs, and 5 rRNA genes.

## GENOME ANNOUNCEMENT

*Mycobacterium* sp. strain 3519A is a rapidly growing scotochromogenic acid-fast bacillus isolated from the sputum of a Cambodian patient with a pulmonary infection. We sequenced and analyzed the whole-genome sequence of strain 3519A in order to describe its genomic content and to determine its phylogenetic relationships for facilitating its detection and identification.

Strain 3519A was cultured on Middlebrook 7H11 agar supplemented with 10% (vol/vol) oleic acid-albumin-dextrose-catalase (Becton, Dickinson, Sparks, MD, USA). The total DNA of strain 3519A^T^ was extracted on the EZ1 biorobot (Qiagen) with an EZ1 DNA tissue kit under a 50-µl elution volume. Total DNA was quantified by a Qubit assay with a high-sensitivity kit (Life Technologies, Inc., Carlsbad, CA, USA) to be 36.2 ng/µl. Genomic DNA was sequenced on a MiSeq platform (Illumina, Inc., San Diego, CA, USA) using both paired-end and mate pair techniques. The index representation for strain 3519A was determined to be 5.51%. A total of 1,116,202 paired-end reads were filtered per the read qualities. The reads were then assembled using the SPAdes software (1). Contigs obtained were combined by use of SSPACE (2) and assisted by manual finishing and GapFiller (3). This yielded a 7,306,349-bp draft genome sequence with a 66.35% GC content, composed of 14 scaffolds and 14 contigs. Open reading frames (ORFs) were predicted using Prodigal (4) with default parameters. Functional annotation was achieved using a BLASTP search against the GenBank database (*E* value, 0.001; coverage, 0.7×; 30% identity) (5) and the Clusters of Orthologous Groups (COGs) database (6). In the case of no hit being found, a second round was done against the NCBI nonredundant protein sequence (NR) database using BLASTP with an *E* value of 1 × 0.001, coverage of 0.7×, and 30% identity. Noncoding genes and miscellaneous features were predicted using RNAmmer (7), ARAGORN (8), Rfam (9), Pfam (10), and Infernal (11). Of the 7,084 predicted genes, 7,029 were protein-coding genes and 55 were RNAs (namely, 3 5S rRNAs, 1 16S rRNA, 1 23S rRNA, and 50 tRNAs). A total of 5,355 genes (76.18%) were assigned putative functions (by a COG or NR BLAST search), 164 genes were identified as ORFans (ORFs with no detectable homology to other ORFs in the database) (2.33%), and 1,295 genes (18.42%) were annotated as hypothetical proteins. The genome of strain 3519A was further incorporated into *in silico* DNA-DNA hybridization (DDH) (12) with genomes selected on the basis of 16S rRNA gene sequence proximity. The DDH values were estimated using the Genome-to-Genome Distance Calculator (GGDC) version 2.0 online tool (13). This analysis yielded a DDH value of 22.60% with *Mycobacterium rutilum* DSM 45405 (GenBank accession number NZ_LT629971), 22.40% with *Mycobacterium holsaticum* M7 (MIGZ00000000), 21.70% with *Mycobacterium rhodesiae* NBB3 (NC_016604), 21.20% with *Mycobacterium chubuense* NBB4 (CP003053), 20.90% with *Mycobacterium mageritense* DSM 44476 (CCBF000000000), and 20% with *Mycobacterium pallens* PYR15 (CP023435).

These data indicate that *Mycobacterium* sp. strain 3519A is related to the *Mycobacterium fortuitum* complex of mycobacteria.

### Accession number(s).

The *Mycobacterium* sp. strain 3519A genome sequence has been deposited at EMBL under the accession number OESG00000000.
